# Superior gastrointestinal visualization and safety of detachable string magnetically controlled capsule endoscopy in patients on antithrombotic agents

**DOI:** 10.3389/fmed.2025.1578285

**Published:** 2025-04-30

**Authors:** Guoyan Bai, Yamei Sun, Zhen Yan, Xue Chen, Hailin Du, Shuwei Li, Jie Zhang

**Affiliations:** Department of Gastroenterology, Beijing Anzhen Hospital, Capital Medical University, Beijing, China

**Keywords:** detachable string magnetically controlled capsule endoscopy, antithrombotic therapy, safety, visualization, endoscopic alternatives

## Abstract

**Background:**

Wireless magnetically controlled capsule endoscopy (WMCCE) offers a non-invasive alternative for patients on antithrombotic therapy, but it has limitations in esophageal visualization and carries risks of capsule retention. We aim to explore the safety and feasibility of detachable string magnetically controlled capsule endoscopy (ds-MCE) for patients on antithrombotic agents.

**Methods:**

This single-center, retrospective study included 387 patients on antithrombotic therapy who underwent magnetically controlled capsule endoscopy between October 2023 and October 2024: 86 with ds-MCE and 301 with WMCCE. Differences in the visualization of the esophagus and stomach, lesions detection and examination time of the esophagus, stomach and small intestine between the two groups were compared, and the safety of ds-MCE was assessed based on string-related discomfort and adverse events. The primary outcome was visualization of the oesophagus. The key secondary outcome was the safety of ds-MCE.

**Results:**

The ds-MCE group achieved significantly higher rates of complete visualization of the esophageal Z-line (52.3% vs. 6.3%, *p* < 0.001) and esophageal mucosa (upper: 87.2% vs. 38.2%; middle: 97.7% vs. 38.9%; lower: 98.8% vs. 53.5%, *p* < 0.001). Detection rates of esophageal lesions, including cancer and varices, were higher in the ds-MCE group (*p* < 0.001). No capsule retention occurred in the ds-MCE group, and 94.2% reported no or mild discomfort.

**Conclusion:**

The ds-MCE improved esophageal visualization and lesion detection compared to WMCCE, offering a safer and less invasive alternative to esophagogastroduodenoscopy for patients on antithrombotic agents with excellent safety and tolerability.

## Introduction

Traditional esophagogastroduodenoscopy (EGD) is invasive and often causes significant discomfort for patients (1, 2). As a result, many patients prefer wireless magnetically controlled capsule endoscopy (WMCCE) for gastrointestinal evaluation. Research has demonstrated that WMCCE can be safely used in high-risk endoscopic populations, without the need to discontinue antithrombotic medications, and offers diagnostic accuracy comparable to EGD (3–5). Despite its advantages, WMCCE has notable limitations, including a restricted ability to thoroughly visualize the esophagus and gastroesophageal junction (6). Additionally, in patients with upper gastrointestinal stenosis caused by conditions such as esophageal diverticula or tumors, capsule retention remains a significant concern, often necessitating invasive endoscopic or surgical interventions for removal (7). These limitations reduce WMCCE’s utility, particularly in high-risk populations such as patients on antithrombotic therapy.

Given the limitations of WMCCE in esophageal visualization and the risks associated with traditional EGD, the development of the detachable string magnetically controlled capsule endoscopy (ds-MCE) offers a promising solution. By enabling precise control and extended observation of esophageal structures, ds-MCE has the potential to improve diagnostic accuracy while ensuring safety, particularly in patients receiving antithrombotic therapy. By incorporating a detachable string, ds-MCE allows for repeated observation of the esophagus through string traction, enabling enables more precise control and visualization of the esophagus and gastroesophageal junction. Additionally, the string provides the ability to retrieve the capsule promptly, mitigating risks associated with capsule retention. Previous studies have demonstrated ds-MCE’s safety and superior diagnostic performance in evaluating upper gastrointestinal diseases among the general population and in patients with liver cirrhosis (8–11).

However, the feasibility and safety of ds-MCE in patients receiving antithrombotic therapy remain unexamined, constituting a significant gap in the current literature. Our study seeks to address this deficiency by evaluating the diagnostic performance of ds-MCE, encompassing esophageal visualization and lesion detection, alongside its tolerability in high-risk patients on antithrombotic therapy.

## Methods

### Study design

This single-center, retrospective study was conducted at Beijing Anzhen Hospital, affiliated with Capital Medical University, from October 1, 2023, to October 27, 2024. All methods were performed in accordance with the relevant STROBE guidelines and regulations, and the study was conducted in accordance with the Declaration of Helsinki. The study protocol was approved by the Ethics Review Committee of Beijing Anzhen Hospital (ethics approval number: 2024112X). The study population included patients receiving antithrombotic therapy who met the indications for endoscopic examination and underwent WMCCE or ds-MCE. All patients agreed and signed written informed consent for the magnetically controlled capsule endoscopy (MCCE) examination. The examination was performed by experienced and qualified endoscopists, each with over 500 MCCE procedures, and the images were independently reviewed by another endoscopist with equivalent experience.

### Participants

The study included patients who underwent MCCE at Beijing Anzhen Hospital, affiliated with Capital Medical University. The inclusion criteria were as follows: patients aged 18 years or older, and those receiving antithrombotic therapy at the time of the procedure. All patients meeting these criteria were included in the analysis, with no additional exclusion criteria applied.

### Study intervention

Patients were classified into two groups: the ds-MCE group for those who underwent ds-MCE, and the control group for those who underwent WMCCE. Basic clinical characteristics were collected for both patient groups, including age, gender, body mass index, gastrointestinal symptom rating scale, antithrombotic therapy details, medical history, and smoking and drinking history. Additionally, the visualization range of esophageal and gastric landmarks, as well as the detection of lesions during MCCE, were recorded. Gastrointestinal injury scores were recorded based on endoscopic findings using the modified gastric Lanza score and the small intestinal injury score system. Furthermore, capsule operation and transit time, capsule ingestion time, string-related discomfort, and any adverse events were also documented.

### The MCE procedure

#### Preparation before examination

After fasting for at least 8 h, all patients were instructed to consume simethicone and pronase granules dissolved in warm water 30 min before the ds-MCE and WMCCE examinations to optimize visualization of the upper gastrointestinal mucosa. Additionally, they were asked to drink 600–800 mL of water 5 min before the examination to expand the gastric cavity.

#### The ds-MCE examination

The ds-MCE system consists of a detachable hollow string and the MCCE system (Ankon Technologies, Wuhan, China). The hollow string is made of sterile, transparent latex and is 120 cm in length. One end has a latex cap, while the other features a tapered connector (9).

The examiner attached the capsule to the latex cap, leaving a small gap, and ensured that it was securely connected to the string and could be detached successfully. The examiner then placed the capsule and part of the string into the patient’s mouth, instructing the patient to swallow the capsule with warm water after a few seconds. The examiner controlled the movement of the capsule by gently pulling on the string, allowing observation of the esophageal mucosa and the Z-line. This process was repeated at least twice. During the procedure, the patient remained seated, with no sedation or local anesthesia required. Afterward, the patient lay on the examination table, and the examiner examined the stomach. Once the capsule entered the stomach, the examiner continued the gastrointestinal examination following standard MCCE procedures (3). To release the capsule, the examiner injected approximately 5 mL of gas into the string using a sterile syringe and gently withdrew the string from the patient’s mouth.

#### The WMCCE examination

After completing the pre-examination preparation, the patient lay on the examination table and swallowed the capsule endoscope with warm water. The examiner then performed the WMCCE according to the standard procedure.

#### Study endpoints

The primary outcome was the visualization of the upper, middle, and lower esophageal mucosa and the esophageal Z-line. The key secondary outcome was safety assessment, including string-related discomfort and adverse events. Other secondary outcomes included visualization of gastric landmarks, lesion detection rates, capsule ingestion time, and esophageal and gastric transit times.

Esophageal and Z-line visualization were evaluated based on the number of quadrants observed during the procedure. Specifically, this study compared the visualization of at least three quadrants (≥75% of the esophageal circumference) and all four quadrants (complete circumference) between the two groups. Gastric landmark visualization was categorized into four levels: “excellent,” “good,” “fair,” and “poor,” where “excellent” was defined as clear observation of 90–100% of the gastric mucosa and “good” as observation of 75–90% (3). This study focused on the ≥ 75 and 90–100% visualization categories.

String-related discomfort was assessed and included capsule ingestion difficulty, pharyngeal discomfort, nausea, vomiting, and coughing. The severity of discomfort was rated on a 4-point scale, from 0 (no discomfort) to 3 (severe discomfort) (10). Adverse events were defined as bleeding due to string traction, aspiration, capsule detachment, retention, separation failure, and string breakage.

### Statistical analysis

Continuous variables with a normal distribution were presented as mean ± SD, and comparisons between groups were performed using the independent-samples t-test. Non-normally distributed continuous variables were presented as median and IQR, and group comparisons were made using the Mann–Whitney U test. Categorical data were described as frequencies or percentages (%), with group comparisons made using the *χ*^2^ test or Fisher’s exact test. All statistical analyses were performed using SPSS 29.0 software, with a *p*-value < 0.05 considered statistically significant.

## Results

### Patient characteristics

From October 1, 2023, to October 27, 2024, 387 participants on antithrombotic therapy underwent MCCE examinations. Of these, 86 participants underwent ds-MCE, and 301 underwent WMCCE. The basic clinical characteristics are presented in Table 1, with no significant differences observed between the two groups.

**Table 1:** Basic clinical characteristics of patients in the ds-MCE and WMCCE groups.

Clinical characteristics	ds-MCE (*n* = 86)	WMCCE (*n* = 301)	*p*-value
Age, mean ± SD, years	65.36 ± 10.44	64.20 ± 10.80	0.38
Sex, *n* (%), male	60 (69.8)	197 (65.4)	0.45
BMI, mean±SD, kg/m^2^	24.55 ± 3.33	24.65 ± 3.45	0.81
GSRS, median (IQR)	1.00 (0–2)	1.00 (0–2)	0.08
Antithrombotic agents, *n* (%)			
Single-antiplatelet drug	37 (43.0)	169 (56.1)	0.16
Dual-antiplatelet drug	34 (39.5)	88 (29.2)	
Anticoagulant drug	9 (10.5)	30 (10.0)	
Antiplatelet and anticoagulation	6 (7.0)	14 (4.7)	
Medical history, *n* (%)			
Hypertension	57 (66.3)	217 (72.1)	0.30
Diabetes	24 (27.9)	113 (37.5)	0.10
Hyperlipidemia	53 (61.6)	190 (63.1)	0.80
Cardiovascular diseases	75 (87.2)	278 (92.4)	0.14
Cerebrovascular diseases	20 (23.3)	59 (19.6)	0.46
Prior peptic ulcer	8 (9.3)	35 (11.6)	0.55
Prior gastrointestinal bleeding	12 (14.0)	25 (8.3)	0.12
Chronic kidney disease	31 (36.0)	93 (30.9)	0.37
Liver cirrhosis	6 (7.0)	8 (2.7)	0.06
Smoking history, *n* (%)	39 (45.3)	119 (39.5)	0.33
Drinking history, *n* (%)	20 (23.3)	95 (31.6)	0.14

The antithrombotic medications include antiplatelet drugs and anticoagulants. The antiplatelet drugs consist of aspirin, indobufen, clopidogrel, and ticagrelor, while the anticoagulants include warfarin, dabigatran, rivaroxaban, and apixaban. BMI, body mass index; GSRS, gastrointestinal symptom rating scale; IQR, interquartile range; SD, Standard Deviation.

#### Esophageal visualization and examination time

**Figure 1:**
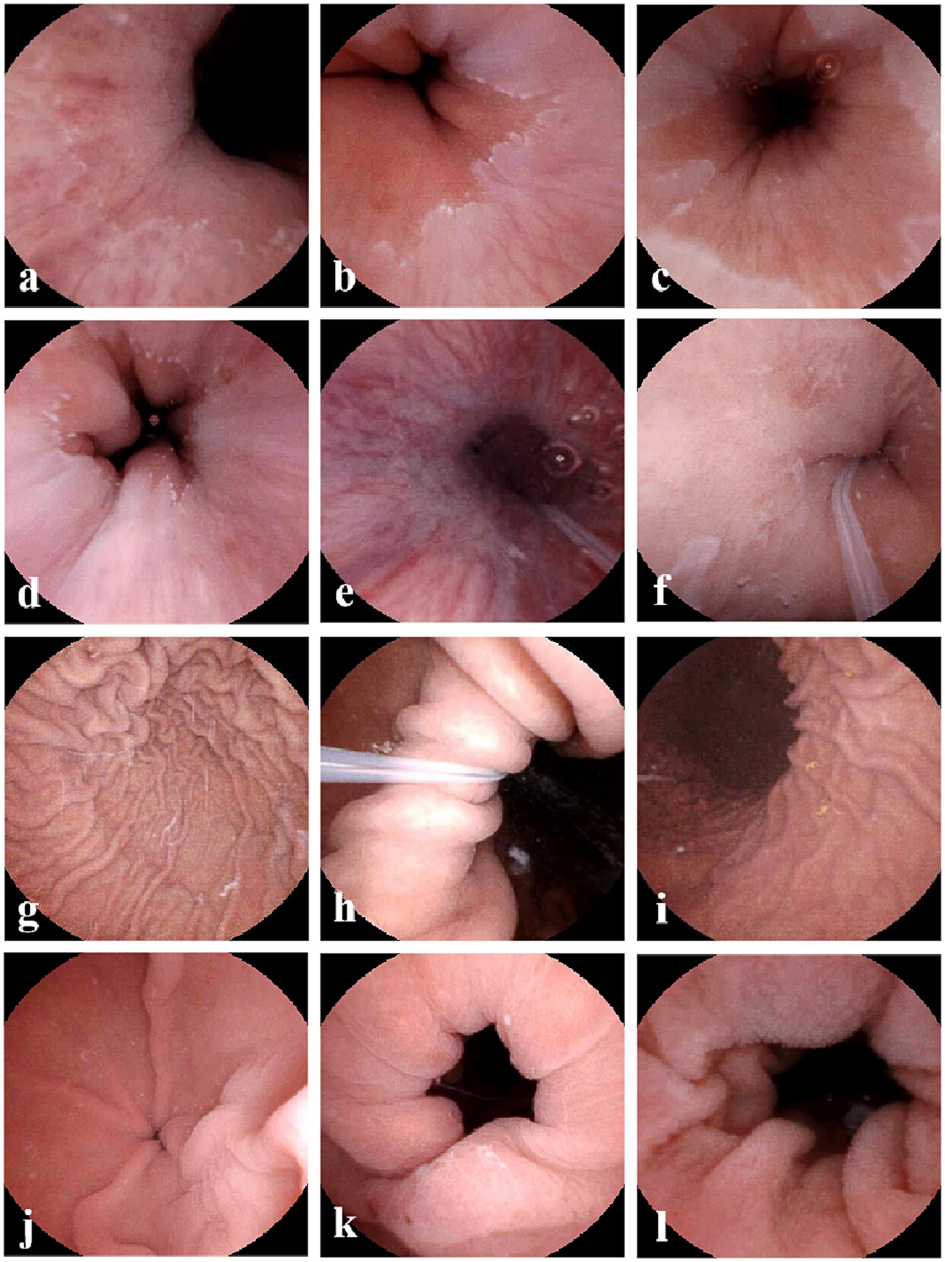
Representative images of the anatomical landmarks by the ds-MCE. **(a)** One-quadrant of the Z-line; **(b)** Two-quadrants of the Z-line; **(c)** Three-quadrants of the Z-line; **(d)** Complete visualization of the Z-line; **(e)** Esophagus; **(f)** Gastric cardia; **(g)** Fundus; **(h)** Body; **(i)** Angulus; **(j)** Antrum; **(k)** Pylorus; **(l)** Small intestinal.

**Figure 2:**
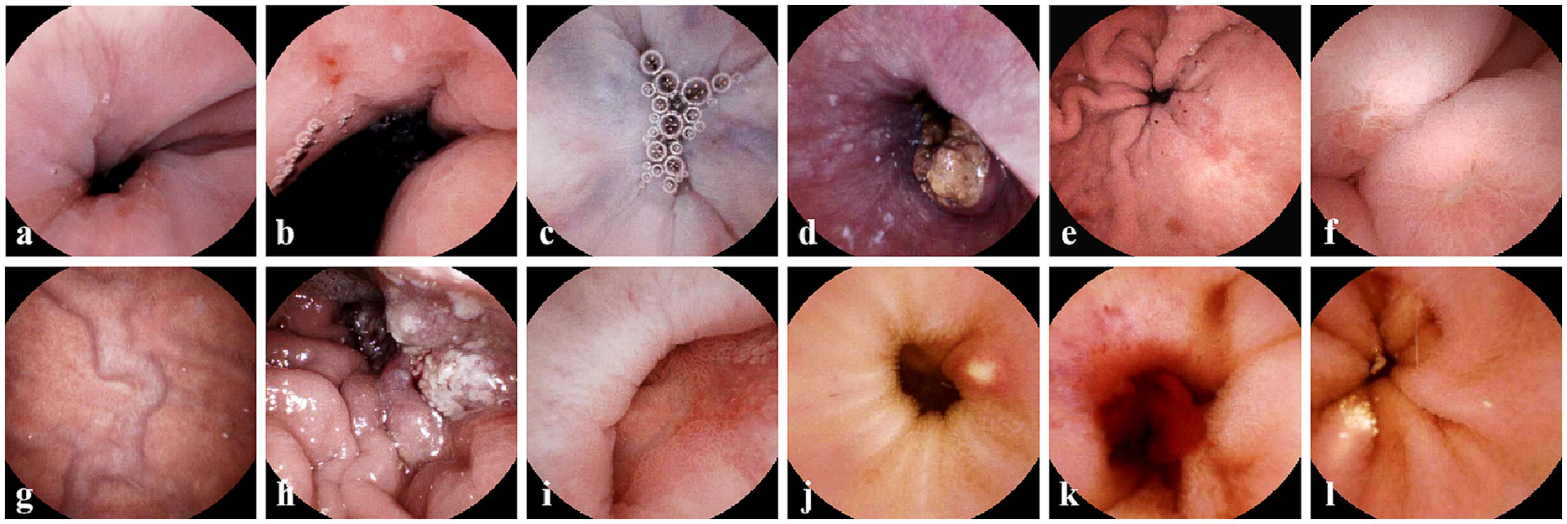
Typical gastric and esophageal lesions by ds-MCE. **(a)** Reflux esophagitis; **(b)** Cardia ulcer and cardiac inflammation; **(c)** Esophageal varices; **(d)** Esophageal cancer; **(e)** Erosive gastritis; **(f)** Gastric ulcer; **(g)** gastric varices; **(h)** Gastric ulcer; **(i)** Duodenal ulcer; **(j)** Small intestinal ulcer; **(k)** Small intestinal bleeding; **(l)** Small intestinal lymphangiectasia.

The ds-MCE significantly improved the visualization of the esophageal Z-line and mucosa compared to the WMCCE (Table 2). Complete visualization of the upper, middle, and lower esophageal mucosa and Z-line in the ds-MCE group was significantly better than in the WMCCE group (87.2% vs. 38.2, 97.7% vs. 38.9, 98.8% vs. 53.5, 52.3% vs. 6.3%, *p*<0.001). The proportions of at least three quadrants observed for the upper, middle, and lower esophageal mucosa and Z-line were higher in the ds-MCE group (96.5% vs. 68.1, 98.8% vs. 65.4, 98.8% vs. 53.5, 73.3% vs. 15.9%, respectively, *p* < 0.001). The capsule ingestion time and esophageal transit time in the ds-MCE group were significantly longer than those in the WMCCE group (*p* < 0.001). Additionally, ds-MCE demonstrated superior diagnostic performance for esophageal diseases compared to WMCCE, with a significantly higher detection rate (*p* < 0.001) (Table 3). Specifically, this ds-MCE identified more cases of cancer, varices, esophagitis, submucosal tumors, and venous dilatation within the study cohort. Representative images of anatomical landmarks and lesions obtained by ds-MCE are shown in Figures 1, 2, respectively.

**Table 2:** The esophageal outcomes and examination time in the ds-MCE and WMCCE group.

Parameters		ds-MCE (*n* = 86)	WMCCE (*n* = 301)	*p* value
Esophageal visualization, *n* (%)				
Upper esophagus	≥3 quadrants	83 (96.5)	205 (68.1)	<0.001
	4 quadrants	75 (87.2)	115 (38.2)	<0.001
Middle esophagus	≥3 quadrants	85 (98.8)	197 (65.4)	<0.001
	4 quadrants	84 (97.7)	117 (38.9)	<0.001
Lower esophagus	≥3 quadrants	85 (98.8)	161 (53.5)	<0.001
	4 quadrants	85 (98.8)	161 (53.5)	<0.001
Z-line	≥3 quadrants	63 (73.3)	48 (15.9)	<0.001
	4 quadrants	45 (52.3)	19 (6.3)	<0.001
Esophageal lesion detection, *n* (%)		40 (46.5)	66 (21.9)	<0.001
Capsule ingestion time, median (IQR), s		29.0 (19.8–42.3)	6.0 (4.0–8.0)	<0.001
Esophagus transit time, median (IQR), s		213.0 (130.0–275.0)	25.0 (9.0–61.5)	<0.001

IQR, interquartile range.

**Table 3:** Number of patients with specific gastrointestinal lesions detected in the ds-MCE and WMCCE groups.

Lesions	ds-MCE (*n* = 86)	WMCCE (*n* = 301)	Total (*n* = 387)	*p* value
Esophagus, *n* (%)				
Cancer	2 (2.3)	0 (0.0)	2 (0.5)	0.05
Varices	3 (3.5)	0 (0.0)	3 (0.8)	0.01
Barrett’s esophagus	4 (4.7)	11 (3.7)	15 (3.9)	0.75
Ulcer	3 (3.5)	2 (0.7)	5 (1.3)	0.85
With bleeding	1 (1.2)	0 (0.0)	1 (0.3)	0.22
Reflux esophagitis	28 (32.6)	52 (17.3)	80 (20.7)	0.003
Submucosal mass	4 (4.7)	1 (0.3)	5 (1.3)	0.01
Venous tumor	4 (4.7)	2 (0.7)	6 (1.6)	0.02
Hiatus hernia	1 (1.2)	4 (1.3)	5 (1.3)	1.00
Stomach, *n* (%)				
Cancer	2 (2.3)	0 (0.0)	2 (0.5)	0.05
With bleeding	1 (1.2)	0 (0.0)	1 (0.3)	0.22
Varices	3 (3.5)	0 (0.0)	3 (0.8)	0.01
Ulcer	30 (34.9)	76 (25.2)	106 (27.4)	0.10
Erosion	81 (94.2)	295 (98.0)	376 (97.2)	0.07
Polyp	9 (10.5)	29 (9.6)	38 (9.8)	0.98
Small intestine, *n* (%)				
Bleeding	1 (1.2)	2 (0.7)	3 (0.8)	0.53
Ulcer	23 (26.7)	67 (22.3)	90 (23.3)	0.47
Erosion	64 (74.4)	223 (74.1)	287 (74.2)	1.00
Polyp	2 (2.3)	6 (2.0)	8 (2.1)	1.00
Lymphangiectasia	13 (15.1)	24 (8.0)	37 (9.6)	0.08
Vascular dysplasia	3 (3.5)	3 (1.0)	6 (1.6)	0.13

All cancer cases were confirmed through subsequent postoperative histopathological examination.

#### Gastrointestinal visualization and examination time

As shown in Table 4, ds-MCE provided better visualization of the gastric cardia compared to WMCCE (*p* < 0.001), whereas no significant differences were observed between the two groups for the gastric fundus, body, angle, antrum, and pylorus. The ds-MCE had a longer gastric operation time compared to WMCCE (*p* < 0.001). In terms of local lesion detection, the ds-MCE group identified more cases of gastric cancer and gastric fundus varices. No significant differences were found between the two groups in terms of gastric and small intestinal lesion detection, gastric modified Lanza score, small intestinal injury score, or gastric and small intestinal transit times during capsule endoscopy.

**Table 4:** The gastrointestinal outcomes of in the ds-MCE and WMCCE group.

Parameters		ds-MCE (*n* = 86)	WMCCE (*n* = 301)	*p*-value
Gastrointestinal visualization, *n* (%)				
Cardia	≥75%	80 (93.0)	227 (75.4)	<0.001
	≥90%	60 (69.8)	135 (44.9)	<0.001
Fundus	≥75%	84 (97.7)	288 (95.7)	0.54
	≥90%	77 (89.5)	246 (81.7)	0.09
Body	≥75%	85 (98.8)	300 (99.7)	0.40
	≥90%	84 (97.7)	293 (97.3)	1.00
Angulus	≥75%	84 (97.7)	299 (99.3)	0.22
	≥90%	82 (95.3)	297 (98.7)	0.08
Antrum	≥75%	85 (98.8)	296 (98.3)	0.59
	≥90%	85 (98.8)	296 (98.3)	0.59
Pylorus	≥75%	84 (97.7)	298 (99.0)	0.31
	≥90%	84 (97.7)	294 (97.7)	1.00
Gastric lesion detection, *n* (%)		84 (97.7)	296 (98.3)	0.65
Small intestinal lesion detection, *n* (%)		70 (81.4)	246 (81.7)	0.94
Gastric modified Lanza score, median (IQR)		2.0 (1.0–4.0)	1.0 (1.0–4.0)	0.14
Small intestinal injury score, median (IQR)		1.0 (1.0–4.0)	1.0 (1.0–3.0)	0.18
Gastric operation time, median (IQR)		21.9 (17.8–26.7)	16.5 (13.4–19.6)	<0.001
Gastric transit time, median (IQR)		75.5 (48.0–110.2)	67.2 (37.5–102.5)	0.17
Small intestinal transit time, median (IQR)		5.7 (4.4–7.6)	5.9 (4.0–7.0)	0.17

IQR, interquartile range.

#### Safety evaluation of the ds-MCE

Among the 86 patients who underwent ds-MCE, the mean discomfort scores associated with string pulling were as follows: ingestion difficulty at 0.31, pharyngeal discomfort at 0.47, nausea at 0.36, vomiting at 0.00, and cough at 0.20 (Table 5). The mean overall discomfort score was 0.56, with 43 patients (50.0%) reporting no discomfort, 38 patients (44.2%) reporting mild discomfort, and five patients (5.8%) reporting moderate discomfort.

**Table 5:** String-related discomfort and adverse events in the ds-MCE group.

Score	0, *n* (%)	1, *n* (%)	2, *n* (%)	3, *n* (%)	Mean score
Ingestion difficulty	65 (75.6)	16 (18.6)	4 (4.6)	1 (1.2)	0.31
Pharyngeal discomfort	47 (54.6)	38 (44.2)	1 (1.2)	0 (0.0)	0.47
Nausea	56 (65.1)	29 (33.7)	1 (1.2)	0 (0.0)	0.36
Vomiting	0 (0.0)	0 (0.0)	0 (0.0)	0 (0.0)	0.00
Cough	69 (80.2)	17 (19.8)	0 (0.0)	0 (0.0)	0.20
Overall discomfort	43 (50.0)	38 (44.2)	5 (5.8)	0 (0.0)	0.56

As for adverse events, none of the patients who underwent ds-MCE experienced bleeding related to string traction, aspiration, capsule detachment, capsule retention, separation failure, or string breakage. In the four patients with gastrointestinal stenosis detected by ds-MCE (including those with esophageal cancer, extrinsic esophageal stricture, and two cases of gastric cancer), the capsule was successfully retrieved using the string, preventing retention. Additionally, in two patients at higher risk of capsule retention, one with a history of small bowel surgery and the other with a large pelvic cancer mass, the capsule was retrieved after completing the gastric examination. However, among the 301 patients who underwent WMCCE, one patient had the capsule embedded in the esophagus due to a stricture at the middle and upper junction caused by compression from the aortic arch. The capsule was subsequently pushed into the stomach via EGD.

## Discussion

Our findings showed that ds-MCE significantly enhanced visualization of esophagus and gastric cardia compared to WMCCE, with superior detection rates of esophageal lesions in patients on antithrombotic therapy. This improvement, likely driven by the detachable string design, allowed for repeated and precise observation of the esophagus, extending the visualization time and enabling close-range imaging of the gastric cardia. These findings align with previous studies in general and cirrhotic populations (8–10, 12), further supporting ds-MCE’s diagnostic value. For gastric visualization, good visualization results were achieved in over 90% of patients in both groups, consistent with previous studies (13–16).

The safety of ds-MCE in patients receiving antithrombotic therapy was confirmed, a population often characterized by advanced age, compromised cardiovascular health, and fragile gastrointestinal mucosa (17, 18). Most patients reported minimal or no discomfort during the procedure, and no severe events such as chest tightness or palpitations occured. Notably, in patients with confirmed or suspected gastrointestinal stenosis, ds-MCE effectively prevented capsule retention by enabling prompt retrieval via the detachable string, eliminating the need for further endoscopic or surgical intervention (19). This expands the indications for MCCE, as current guidelines contraindicate WMCCE for patients with gastrointestinal obstruction or stenosis (3). By mitigating retention risks, ds-MCE offers a safe alternative for these patients.

For patients with suspected gastrointestinal bleeding, such as those with melena or positive fecal occult blood tests, the superior visualization of the esophagus, stomach, and small intestine with ds-MCE enables accurate assessment of the extent and location of gastrointestinal injury. This allows clinicians to assess bleeding status accurately, adjust antithrombotic medications as needed, and make informed treatment decisions, thereby preventing embolic or bleeding events due to premature discontinuing antithrombotic therapy or excessive antithrombotic therapy. Additionally, with its superior esophageal visualization and ability to prevent capsule retention, ds-MCE offers a better-tolerated examination option for patients with obvious esophageal symptoms or suspected esophageal disease, especially those with cirrhosis requiring variceal screening, imaging evidence of gastrointestinal stenosis, or suspected gastrointestinal tumors (20, 21). Although the higher detection rates of cancerous and variceal lesions observed in the ds-MCE group may partially reflect selection bias, they nonrtheless underscore ds-MCE’s unique capability to safely and accurately identify critical esophageal lesions that are challenging to detect with WMCCE.

Our findings have several important clinical implications for patients with cardiovascular disease on antithrombotic therapy. The improved diagnostic yield and favorable safety profile of ds-MCE support its use as a well-tolerated modality for comprehensive evaluation of the esophagus, stomach, and small intestine, particularly in patients with increased bleeding risk or poor tolerance to conventional EGD. It also enables the safe assessment of patients with suspected gastrointestinal stenosis or a history of capsule retention, thereby expanding the population eligible for capsule-based endoscopic evaluation. Moreover, its accurate visualization and lesion detection can directly inform clinical management decisions, including the optimization of antithrombotic therapy, potentially reducing the risk of thrombotic and bleeding complications. Overall, these advantages underscore the value of ds-MCE as a feasible and patient-friendly diagnostic modality and support its integration into routine clinical workflows for patients on antithrombotic therapy. In the future, ds-MCE may be considered a preferred option for cardiovascular patients on antithrombotic agents, particularly when conventional endoscopy is contraindicated or poorly tolerated.

We must acknowledge that our study has several limitations. Firstly, the single-center retrospective design may limit the robustness of the results and affect external validity. Future validation through multicenter studies is recommended. Secondly, the relatively small sample size, particularly in the ds-MCE group, may restrict the statistical power to detect differences in rare adverse events or specific subpopulations. Thirdly, the longer esophageal and gastric transit time observed with ds-MCE may pose challenges for less experienced operators, requiring additional training to optimize its application. Future studies should focus on multicenter, prospective trials with larger and more diverse cohorts to validate these findings and explore the long-term clinical impact of ds-MCE in high-risk populations. Lastly, although ds-MCE offers excellent visualization and a favorable safety profile, it currently lacks the ability to perform biopsies and therapeutic interventions, which remains one of the major limitations of any MCCE compared to traditional EGD. The clinical translation of MCCE equipped for both biopsy and therapeutic interventions holds promise for demonstrating enhanced clinical value in patients on antithrombotic therapy (22).

In conclusion, compared to WMCCE, ds-MCE is a safer and more effective endoscopic approach for patients on antithrombotic therapy, significantly improving esophageal visualization and lesion detection while minimizing the risk of capsule retention. The study highlights its clinical potential to optimize antithrombotic management and improve outcomes in high-risk patients, laying the foundation for broader clinical application.

## Data Availability

The original contributions presented in the study are included in the article/supplementary material, further inquiries can be directed to the corresponding author.
